# Water Bacterial and Fungal Community Compositions Associated with Urban Lakes, Xi’an, China

**DOI:** 10.3390/ijerph15030469

**Published:** 2018-03-07

**Authors:** Haihan Zhang, Yue Wang, Shengnan Chen, Zhenfang Zhao, Ji Feng, Zhonghui Zhang, Kuanyu Lu, Jingyu Jia

**Affiliations:** 1 School of Environmental and Municipal Engineering, Xi’an University of Architecture and Technology, Xi’an 710055, China; yuewang_17@163.com (Y.W.); chenshengnan@xauat.edu.cn (S.C.); zhenfangzhao@163.com (Z.Z.); fengji0423@163.com (J.F.); Zhangzhonghui1702@163.com (Z.Z.); lukuanyu@163.com (K.L.); jiajingyuxiann@163.com (J.J.); 2 Institute of Environmental Microbial Technology, Xi’an University of Architecture and Technology, Xi’an 710055, China

**Keywords:** urban lake, water quality, microbial community composition, Illumina Miseq sequence

## Abstract

Urban lakes play a vital role in the sustainable development of urbanized areas. In this freshwater ecosystem, massive microbial communities can drive the recycling of nutrients and regulate the water quality. However, water bacterial and fungal communities in the urban lakes are not well understood. In the present work, scanning electron microscopy (SEM) was combined with community level physiological profiles (CLPPs) and Illumina Miseq sequence techniques to determine the diversity and composition of the water bacterial and fungal community in three urban lakes, namely Xingqing lake (LX), Geming lake (LG) and Lianhu lake (LL), located in Xi’an City (Shaanxi Province, China). The results showed that these three lakes were eutrophic water bodies. The highest total nitrogen (TN) was observed in LL, with a value of 12.1 mg/L, which is 2 times higher than that of LG. The permanganate index (COD_Mn_) concentrations were 21.6 mg/L, 35.4 mg/L and 28.8 mg/L in LG, LL and LX, respectively (*p* < 0.01). Based on the CLPPs test, the results demonstrated that water bacterial communities in the LL and LX urban lakes had higher carbon source utilization ability. A total of 62,742 and 55,346 high quality reads were grouped into 894 and 305 operational taxonomic units (OTUs) for bacterial and fungal communities, respectively. Water bacterial and fungal community was distributed across 14 and 6 phyla. The most common phyla were Proteobacteriaand Cyanobacteria. Cryptomycota was particularly dominant in LL, while Chytridiomycota and Entomophthormycota were the most abundant fungal phyla, accounting for 95% of the population in the LL and 56% in the LG. Heat map and redundancy analysis (RDA) highlighted the dramatic differences of water bacterial communities among three urban lakes. Meanwhile, the profiles of fungal communities were significantly correlated with the water quality parameters (e.g., COD_Mn_ and total nitrogen, TN). Several microbes (*Legionella* sp. and *Streptococcus* sp.) related to human diseases, such as infectious diseases, were also found. The results from this study provides useful information related to the water quality and microbial community compositions harbored in the aquatic ecosystems of urban lakes.

## 1. Introduction

Urban lakes play a pivotal role in reclaimed water resource utilization and the development of urban environments [1]. In the past few decades, an increasing number of man-made urban lakes used for recreation have been built around city parks in order to increase the quality of the living environment for citizens [2,3]. Recently, urbanization has been improved due to the rapid industrialization in developing countries [4]. However, numerous quantities of pollutants from industrial and human activities are frequently discharged into the lakes [1,5]. Therefore, urbanization has a detrimental influence on the water body through pollution discharge and contribution to climate change [4,6]. Interestingly, Xu et al. [6] studied the relationship between urbanization-related factors and bacterial communities in the soil of urban park. This study revealed that urbanization had a dramatic effect on the composition of the bacterial community in urban park soils. Heavy metal and microplastics in the surface soil around the urban lake can be flushed into the water body during the storm events [5,7]. The water quality and biological characteristics of unban lake will be influenced, with water quality deterioration and algal bloom frequently occurring [3,5,7]. Unfortunately, the characteristics of water quality and water microbial community living in urban lake ecosystems are not well understood because urbanization can shape the diversity of environmental bacterial communities [6].

The diversity of aquatic microbial communities has been widely investigated using a combination of culture-dependent and molecular techniques [8,9,10]. A culture-dependent method named BIOLOG was employed to investigate the functional diversity of water bacterial communities based on the community level physiological profiles (CLPPs) [8,9]. With the development of next generation sequencing technique (NGST) and bioinformatics, high-throughput Illumina Miseq sequencing has greatly improved our understanding of microbial communities living in water and sediment environmental conditions [11]. During the past few decades, multiple works have been undertaken, which examine the microbial community associated with freshwater ecosystems [7,8,9,10,11,12]. These ecosystems have also been compared to massive reports focused on revealing the water microbial communities in urban water supply reservoirs [8,9,11,12], polluted urban rivers [13] and wastewater treatment systems [14]. Few studies have focused on examining the water bacterial and fungal community structure associated with urban lakes, especially in regions of water shortage as well as arid and semi-arid areas of developing countries.

Water quality can be regulated by microbe metabolism and complex microbial compositions due to microbes carrying out most of the essential biogeochemical process in urban lakes, such as iron, sulfur, phosphorus and nitrogen recycling [1,3,5]. Almeida-Dalme et al. [15] investigated the water bacterial and archaeal communities in the north arm of Great Salt Lake (Utah, United States), and demonstrated that the seasonal dynamics of community diversity was influenced by lake elevation and salinity. Recently, Morrison et al. [16] conducted a spatiotemporal survey of the water microbial communities in Grand Lake and found that bacterial communities could mediate organic matter recycling and regulate algal bloom in this stratified lake. Rosińska et al. [17] found that algal bloom and cyanobacteria species changed dramatically before and during the restoration process of the heavily polluted Swarzędzkie Lake, which suggested that higher water temperature stimulated cyanobacteria growth. However, the characteristics of water fungal community structure and composition in urban lake ecosystems remains unclear.

Therefore, the general objective of this work is to explore the water quality and microbial community diversity associated with urban lakes, including Xingqing lake (LX), Geming lake (LG) and Lianhu lake (LL), located in Xi’an City (Shaanxi Province, China). The specific aims of present study are: (1) to assess the general water quality parameters; (2) to reveal morphological features of water microbial community using scanning electron microscopy (SEM); (3) to determine the functional diversity of water bacterial communities based on community level physiological profiles (CLPPs) as well as water bacterial and fungal community compositions using 16S rRNA and Internal Transcribed Spacer (ITS) genes in the Illumina Miseq sequence in LX, LG and LL urban lakes; and (4) to investigate the relationship between water quality and microbial community structure. This study will improve the foundational database for evaluating the biological characteristics of water quality and enhance assessments of the urban lake ecosystem inhabitants.

## 2. Materials and Methods

### 2.1. Study Site Description

The study site is located in Xi’an City, Shaanxi Province, in northwest China. Xi’an is the capital city of Shaanxi Province, which is one of the most important cradles of Chinese civilization. It marked the start of the famous “Silk Road”, which linked China with central Asia and the Roman Empire [18]. It served as the first capital of a unified China and periodically as capital of 11 dynasties. Xi’an City is geographically located in the center of the fertile Guanzhong Plain, which is surrounded by the Qinling Mountains to the south and the Wei River in the north [19]. The average annual precipitation is approximately 450 mm. The temperature varies from a low of −10 °C in January to a high of 38 °C in August [18,19]. In order to improve the environmental quality, several urban lakes have been built [18]. However, the water quality of these urban lakes has decreased due to excessive richness of nutrients in the lake, while eutrophication and algal blooms occur during the warm summer months [18]. During the past few years, we systematically investigated the water denitrifying bacterial community structure in lakes [18] and urban source water reservoirs [8,9,11,12,20]. Several effective denitrifying bacterial strains were isolated from sediments [21], which can be used for bioremediation of polluted water in urban lake. To fully understand the water microbial community composition of urban lakes in Xi’an City, Xingqing lake (LX), Geming lake (LG) and Lianhu lake (LL) are investigated in the present study, which are the typical and oldest lakes in Xi’an. The detailed information about lakes are listed in Table 1.

### 2.2. Sampling Process

The sampling process was conducted in June 2014. As previously described by Kang et al. [18,21], three representative sampling sites were selected in each urban lake. Water samples were collected at a depth of 0.5 m in each urban lake with sterile plastic containers [18], stored in ice coolers and transported to the laboratory in Institute of Environmental Microbial Technology, Xi’an University of Architecture and Technology (IEMT-XAUAT) within 4 h. One part of water sample was used for determining the water physicochemical parameters and bacterial community level physiological profiles (CLPPs) immediately. The rest of the water samples were used for examination of water bacterial and fungal communities. For these samples, the water was filtered using polycarbonate membrane (0.22 μm) [18] and stored at −20 °C for determination of water microbial communities.

### 2.3. Water Physicochemical Analysis

To determine the water quality parameters, water temperature, dissolved oxygen (DO) and pH were determined in situ using the thermometer, portable dissolved oxygen meter and pH meter (Hach, Loveland, CO, USA) [18]. Meanwhile, the concentrations of total nitrogen (TN), ammonia nitrogen (NH_4_^+^-N), nitrate nitrogen (NO_3_^−^-N) and total phosphorus (TP) were spectrophotometrically examined using a continuous flow analyzer (Seal Analytical AA3, Norderstedt, Germany) after the water samples were filtered by a polycarbonate membrane (0.22 μm) [22]. Permanganate index (COD_Mn_) was measured using spectrophotometer with the DR6000 (Hach, Loveland, CO, USA). Fe concentration was determined by inductively coupled plasma-mass spectrometry (ICP-MS) (Perkin Elmer, Norwalk, CT, USA) [22].

### 2.4. Scanning Electron Microscopy (SEM) Determination

To view the image of water microbial communities, scanning electron microscopy (SEM) was used. Briefly, the water microbial community samples were collected on the membrane surface (0.22 μm, Millipore), before the glutaraldehyde fixative solution and ethanol were added for dehydration. After this, we sprayed the sample with gold-palladium, before installing the sample onto the sample holder using SEM (JSM-6510LV, Jeol, Tokyo, Japan) for the photograph to explore the microstructure and surface morphology of water microbes, especially algal cells [23].

### 2.5. Determination of Water Microbial Community Functional Diversity

To determine the functional diversity of water bacterial communities, community level physiological profiles (CLPPs) technique named BIOLOG was used to explore metabolic fingerprints of the water bacterial communities [8]. To examine the functional diversity of water bacterial communities, ECO plates were selected. A total of 31 sole carbon sources were located in each ECO plate [9]. In the clean workbench, 150 μL of the water samples was added into each hole of ECO plate using eight electronic pipettes (Eppendorf, Hamburg, Germany). The inoculated ECO plates were put into the chamber and incubated at 28 °C in darkness [9]. The carbon source utilization (amines, amino acids, carbohydrate, carboxylic acid, phenolic compound and polymers) were monitored at intervals of 24 h for 10 days at an optic density (OD) of 590 nm (BIOLOG, Hayward, CA, USA) [11]. The average well color development (*AWCD*_590nm_), Richness diversity (*R*), Shannon’s diversity (*H’*) and carbon source utilization were calculated based on 96 h of incubation data, with these indices calculated as previously described [8,9,11]. The assays were performed in triplicate (*n* = 3).

### 2.6. Water Microbial Community DNA Extraction

To extract total water microbial genomic DNA, 500-mL water samples were filtered onto polycarbonate membrane filters (0.22 µm), before the microbial DNA was extracted using a Water DNA Kits (Omega Bio-tek, Palo Alto, CA, USA). DNA samples were further purified using a DNA Purification Kits (Thermo Fisher Scientific, Waltham, MA, USA) following the manufacturer’s protocols. Purified DNA was stored at −20 °C [11].

### 2.7. Determination of the Water Bacterial and Fungal Community Compositions

To explore the water microbial community structure, Illumina Miseq sequence method was used [24]. In the present study, water bacterial and fungal communities were determined. For water bacterial community examination, the specific bacterial primers of 515-f (5′-GTGCCAGCMGCCGC GG-3′) and 907-r (5′-CCGTCAATTCMTTTRAGTTT-3′) were used in the amplification of the 16S rRNA genes [25]. For water fungal community determination, the internal transcribed spacer (ITS) gene region was amplified by primers ITS1-f (5′-CTTGGTCATTTAGAGGAAGTAA-3′) and ITS2-r (5′-GCTGCGTTCTTCATCGATGC-3′) [26]. The 20-μL mixture in each PCR tube contained 10 ng of the DNA template, 2 μL of dNTP (2.5 mM), 4 μL of 5×FastPfu Buffer, 0.8 μL of each forward and reverse primers (5 μM), 0.4 μL of FastPfu Polymerase (Thermo Fisher Scientific, Waltham, MA, USA) and a balance of ddH_2_O. The PCR amplification program was carried out under the following conditions: 95 °C for 5 min, followed by 27 cycles of denaturation at 95 °C for 30 s; and annealing at 55 °C for 30 s and extension at 72 °C for 45 s. A final extension phase was performed at 72 °C for 10 min and 12 °C until halted from a PCR thermal cycler (C-1000, Bio-Rad, Hercules, CA, USA). The PCR product samples were checked using 1.5% agarose gel electrophoresis (Bio-Rad), before being purified by a PCR product purification kit (Thermo Fisher Scientific, Waltham, MA, USA) according to the manufacturer’s instructions. The concentration of purified PCR product was determined using a Nano Drop™ 2000 (Thermo Fisher Scientific, Waltham, MA, USA). Amplicons were subsequently sent to Shanghai Majorbio Bio Pharm Technology Co., Ltd. (Shanghai, China) and sequenced using the Illumina Miseq high through put sequencing platform.

### 2.8. Nucleotide Sequence Accession Number

The 16S rRNA gene and ITS sequences derived from Illumina Miseq data were deposited at the National Center for Biotechnology Information (NCBI) database (https://www.ncbi.nlm.nih.gov/) with the accession number SRP044894.

### 2.9. Data Analysis

To test the significant difference of water quality parameters among three urban lakes, one-way analysis of variance (ANOVA) was used and followed by a Tukey HSD post-hoc test (SPSS 22.0, SPSS Inc., Chicago, IL, USA). For BIOLOG data, *AWCD*_590nm_, Richness diversity (*R*) and Shannon’s diversity (*H’*) were calculated as described by Zhang et al. [8,11] and Yang et al. [9]. One-way ANOVA was used to determine differences among urban lakes. As previously described [11], quantitative insights into microbial ecology (QIIME) (http://bio.cug.edu.cn/qiime/) was used to analyze Ilumina Miseq sequence data [25]. Specifically, primers were trimmed and the low-quality sequences were excluded if the length was less than 35 bases [26]. Operational taxonomic units (OTUs) grouped at the significance level of 0.97 were taxonomically assigned using the Ribosomal Database Project (RDP) classifier PyNastat a 50% bootstrap confidence level (http://rdp.cme.msu.edu/classifier/classifier.jsp). Water fungal OTUs were taxonomically assigned following the databases of Fungorum (http://www.indexfungorum.org/) and MycoBank (http://www.mycobank.org/). Reads that did not match any microbial sequences were identified as unclassified. The alpha diversity of bacterial and fungal communities was measured using abundance-based coverage estimators (ACE), *Chao*1, Simpson diversity (*D*) and Shannon’s diversity (*H’*) indices (MOTHUR software version 1.22.2, The University of Michigan, Ann Arbor, MI, USA, http://www.mothur.org). Heat map fingerprints of representative bacterial and fungal community structure at genus level were performed using R software (version 3.2.3, Lincoln, NE, USA). Redundancy analysis (RDA) was employed to build models explaining relationships between water quality and water microbial community structure. The statistical significance in the regression was evaluated using Monte Carlo based permutation test (MCP) based on a significance level of 5% [27]. Multivariate statistics for microbial community data was performed by CANOCO 5 for Windows statistical packages (version 5.02, Wageningen, The Netherlands, http://www.canoco5.com/) [28].

## 3. Results and Discussion

### 3.1. Water Quality Parameters

Water physicochemical properties in urban lakes are shown in Table 2. There were no significant differences in water temperature (*p >* 0.05) and pH value (*p >* 0.05) among the LG, LL, and LX urban lakes. Dissolved oxygen (DO) of the lake water was in the range of 8.3–10.2 mg/L. The nitrate concentration in LG was 5.3 mg/L, which was significantly higher than that of LX (*p <* 0.001) (Table 2). The highest total nitrogen (TN) was observed in LL with a value of 12.1 mg/L, which was 2 times higher than that of LG (*p <* 0.01). However, total phosphorus (TP) in LG was 0.21 mg/L. The COD_Mn_ concentrations were 21.6 mg/L, 35.4 mg/L and 28.8 mg/L in LG, LL and LX, respectively (*p <* 0.01) (Table 2). The lowest Fe concentration was observed in LX with a value of 0.01 mg/L (*p <* 0.01) (Table 2). The only source of water in these lake is the reclaimed water resource from the wastewater treatment plant in Xi’an City. The drainage waters are high in TN and organic carbon, which can induce algal bloom during the summer season. In Xi’an City, the urban expansion around the lake began in the 1980s, with a large amount of wastewater having already been discharged into the lakes. The water quality in other urban lakes was monitored by Kang et al. [18], who found that the highest TN in Fengqing lake was 6.5 mg/L, while the COD_Mn_ was 13.12 mg/L. A similar study conducted by Kozak et al. [29] showed a TN of 4 mg/L, NO_3_^−^-N of 1.9 mg/L and a TP of 0.08 mg/L in the urban Lake Głębokie, Poland. The nutrients in the urban lakes of Xi’an City had higher levels than that of the largest freshwater lake in China, Lake Poyang, where the water quality was assessed using eutrophication and ecological indicators [30]. In order to improve the water quality, several restoration efforts can be conducted, such as removing the nutrient-laden sediments and manipulation of the fish population [31]. A large number of urban lakes suffer from algal bloom due to the endogenous N and P released from the sediment, especially in warm summer months [3]. Cyanobacteria blooms are associated with offensive odors, while the water-soluble neurotoxins are released from cyanobacterial cells [18,31,32]. Neurotoxins are harmful to children playing with water of urban lakes, because the main function of urban lakes is entertainment for citizens, such as recreational boating activities. It is suggested that the water quality in urban lake has significant influence on microbial community structure.

### 3.2. Scanning Electron Microscopy (SEM) Images

**Figure 1:**
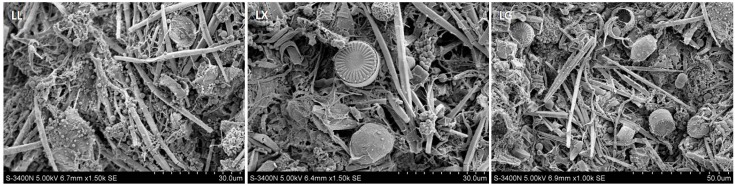
Scanning electron microscopy (SEM) images of water microbial community composition associated with Lianhu lake (LL), Xingqing lake (LX), and Geming lake (LG) in Xi’an City, Shaanxi Province, China.

As shown in Figure 1, based on the SEM examination, a large number of spherical bacteria and algae were found to live in the studied lakes. *Cyclotella* sp. (diameter of 15–20 μm) and *Closteium* sp. (length of 10–20 μm) were found in LX. LX was dominated by *Chlorella* sp. Meanwhile, *Synedra* sp. (length of 50 μm) and *Cyclotella* sp. (diameter of 15–20 μm) were observed in LG (Figure 1). An artificial urban lake next to Louisiana State University has a water quality problem due to algal blooms. Identification of algal communities suggested that Bacillariophyceae, Cyanophyceae and Chlorophyceae were the dominate phyla [31]. More than 100 storm drains currently discharge into the lake, which can cause serious algal bloom with higher *Chl a* concentration. This result is different from previous studies, which have suggested that cyanobacteria blooms are popular in eutrophication lakes. This may be due to different urban lakes having different water quality, which can shape the structure and composition of algal communities.

### 3.3. Functional Diversity of Water Bacterial Communities

The carbon source utilization patterns (functional metabolic diversity) of water bacterial communities are shown in Table 3. *AWCD*_590nm_ was usually conducted to measure the metabolic activity of bacterial communities by reflecting carbon substrate utilization performance [8]. The highest *AWCD*_590nm_ was observed in LL, while the lowest *AWCD*_590nm_ existed in the LG (*p <* 0.01). Assessment of the functional diversity of water bacterial communities in lakes can be useful for detecting the self-purification ability of water bodies [8,9]. Richness diversity (*R*) and Shannon’s diversity (*H’*) were higher in LL. Furthermore, utilization of the six types of carbon sources changed significantly among different urban lakes (Table 2). The lowest utilization of amino acids was observed in LG (*p <* 0.001). Interestingly, Yang et al. [9] used the BIOLOG method to explore the water bacterial functional diversity in an urban drinking water reservoir and revealed that *AWCD*_590nm_ values were in the range of 0.38–0.62, which was lower than that of LL and LX. This indicated that the water bacterial communities in the LL and LX urban lakes had higher carbon source utilization ability. Community-level physiological profiles (CLPPs) tests have been previously employed to determine differences in metabolic fingerprints in bacterial communities in the water [8] and sediment [12] of urban water source reservoir and springs [33]. Similarly, a study conducted by Gordon-Bradley et al. [33] also suggested that carbon sources utilization profiles of freshwater spring water bacterial communities during blooming and non-blooming stages changed significantly, while carbohydrates and polymers were most utilized during the non-blooming stage.

### 3.4. Water Bacterial and Fungal Community Compositions

**Figure 2:**
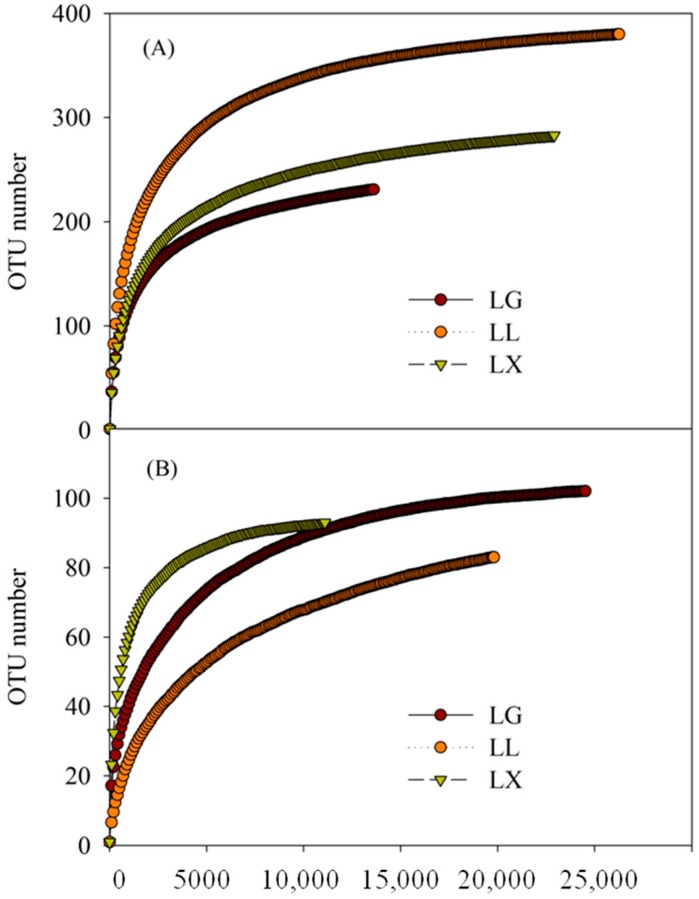
Water bacterial (**A**) and fungal (**B**) community operational taxonomic units (OTUs) number at 0.97 level and the reads number sampled based on Illumina Miseq data of Geming lake (LG), Lianhu lake (LL), and Xingqing lake (LX) in Xi’an City, Shaanxi Province, China.

After high throughput sequencing analysis, based on the 16S rRNA gene sequencing from Illumina Miseq platform of water bacterial communities, a total of 62,742 reads across the three samples were passed through the high-quality filters with an average read length of 400 bp (Table 4). In total, there were 894 different operational taxonomic units (OTUs) for bacterial communities with 97% sequence similarity, with the rarefaction curves of the OTUs number for each read sampled shown in Figure 2. For bacterial communities, the abundance-based coverage estimator (ACE) was in the range of 254–393. The highest *Chao*1 index was observed in LL, which was 53.3% higher than that of LG. However, the lowest Shannon diversity (*H’*) was found in LX with 3.48 (Table 4). Meanwhile, for water fungal communities, 55,346 high-quality reads were obtained after removing low-quality reads (Table 4). These sequences were grouped into 305 different OTUs with97% sequence similarity. Shannon diversity (*H’*) in LX was 2.59, which was 2.9 times higher than that of LL. ACE index did not vary greatly between LG and LL. *Chao*1 was generally higher in LG, compared with LL and LX, with an average value of 98. Overall, the Simpson diversity index (*D*) of bacterial community was lower than that of fungal community from three urban lakes.

**Figure 3:**
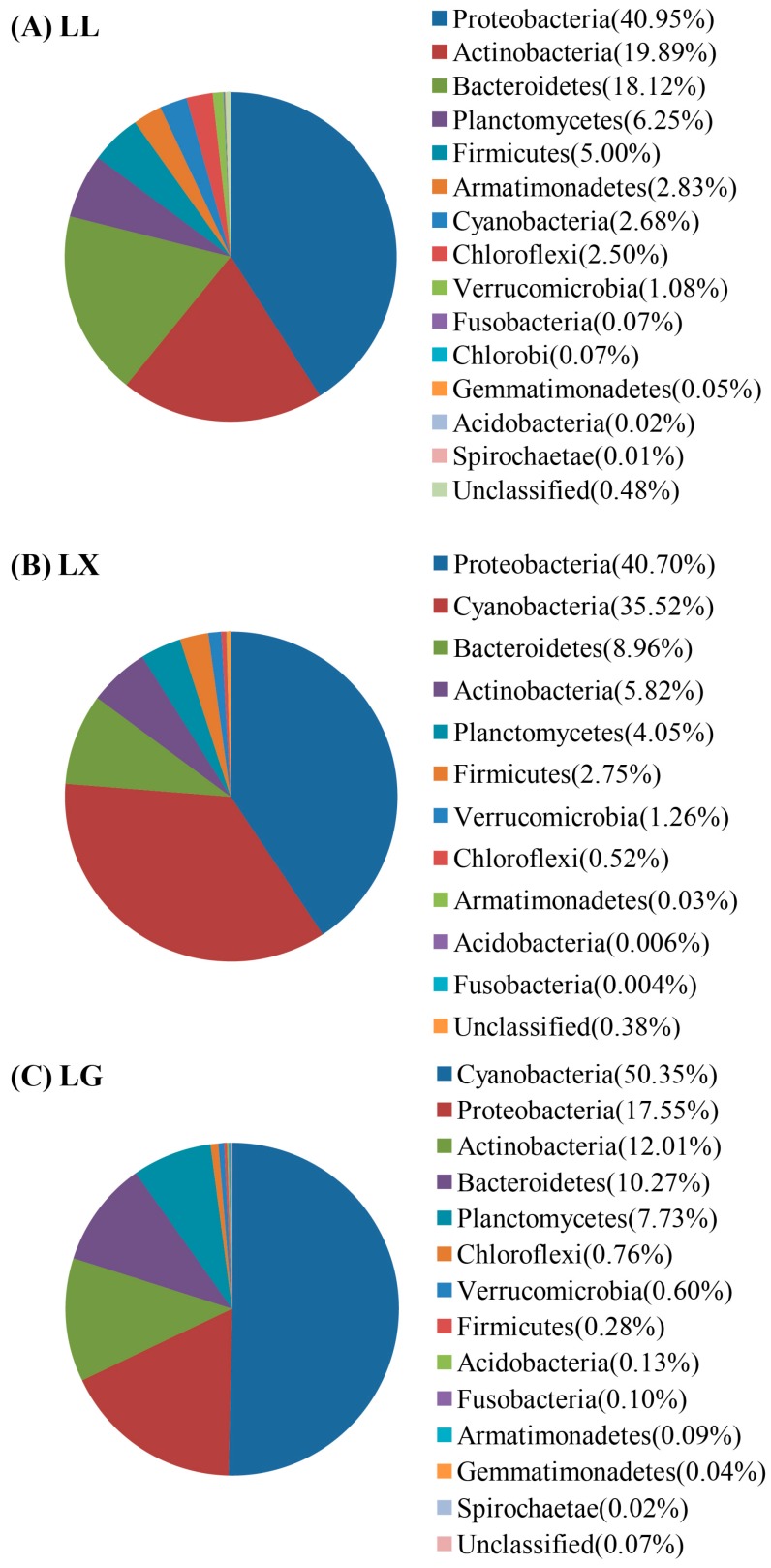
Taxonomic classification of water bacterial community reads of Illumina Miseq sequencing data of (**A**) Lianhu lake (LL), (**B**) Xingqing lake (LX), and (**C**) Geming lake (LG) into phylum levels using the Ribosomal Database Project (RDP) classifier.

To further examine the water bacterial community structure, the taxonomic classification of water bacterial community in phylum levels was performed, with the results shown in Figure 3. The lake water bacterial community was distributed among 14 different phyla including Proteobacteria, Actinobacteria, Bactroidetes, Planctomycetes, Firmicutes, Armatimonadetes, Cyanobacteria, Chloroflexi, Verrucomicrobia, Fusobacteria, Chlorobi, Gemmatimonadetes, Acidobacteria and Spirochaeate. The most common phyla in LL were Proteobacteria (40.95%), Actinobacteria (19.89%) and Bacteroidetes (18.12%). Meanwhile, Proteobacteria (40.70%) and Cyanobacteria (35.52%) were dominant in LX, compared to all classifiable 16S rRNA sequences. A small proportion of sequences (0.38%) was unclassified. Approximately 68% of reads could be classified with Cyanobacteria (50.35%) and Proteobacteria (17.55%) in LG (Figure 3). Interestingly, the Cyanobacteria was the second and first most dominant phylum in LX and LG, respectively (Figure 3). This is consistent with the SEM results. This is consistent with related reports [8,9], which suggested that Proteobacteria was the most dominant phyla in reservoirs [11,12] and other environmental conditions [6]. Bacteroidetes, Proteobacteria, and Firmicutes have been also observed in wastewater-influenced lakes [34].

**Figure 4:**
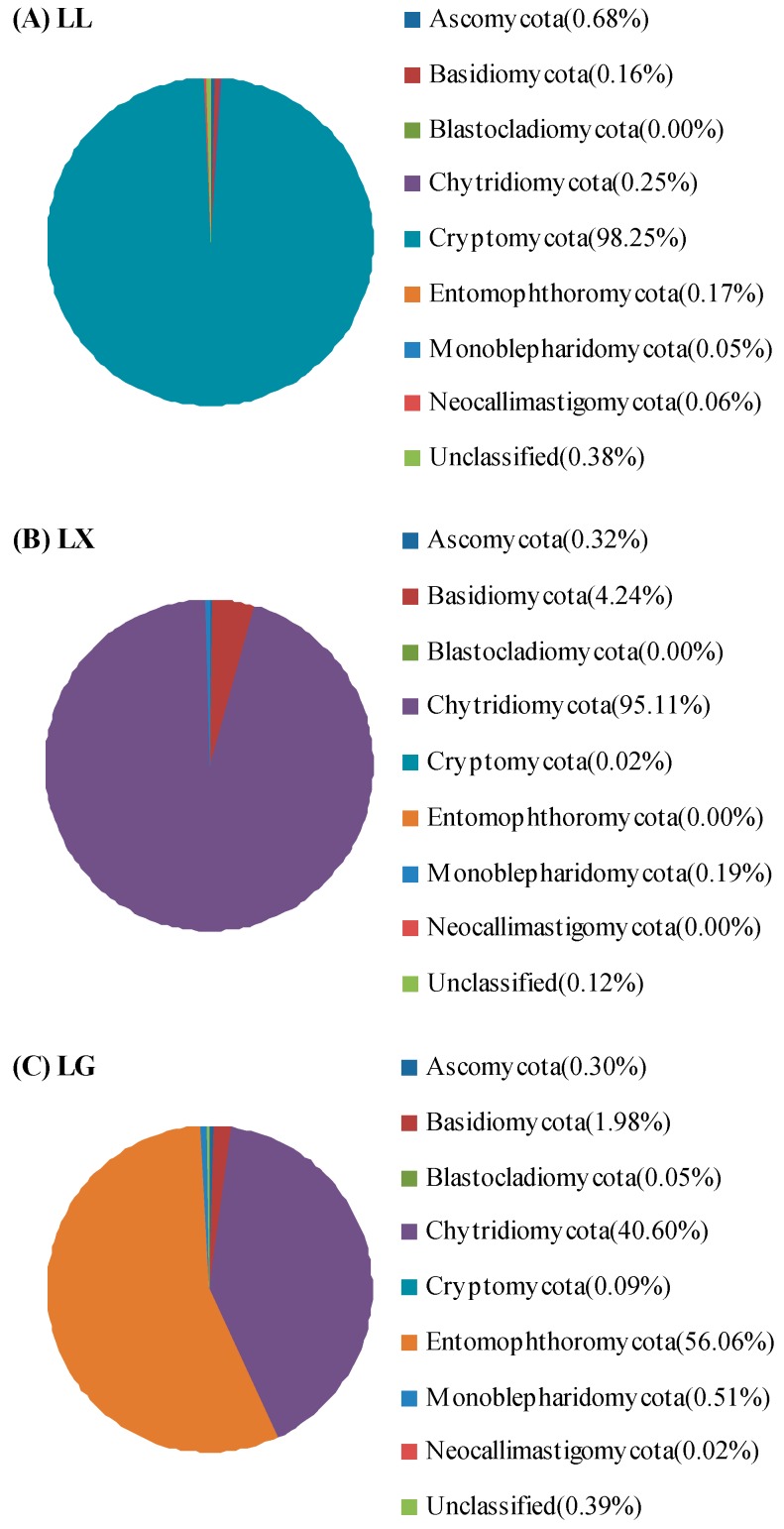
Taxonomic classification of water fungal community reads of Illumina Miseq data of (**A**) Lianhu lake (LL), (**B**) Xingqing lake (LX), and (**C**) Geming lake (LG) into phylum levels using the UNITE (http://unite.ut.ee) classifier database.

To explore the fungal community reads of Illumina Miseq sequence, the UNITE database (http://unite.ut.ee) classifier and NCBI Taxonomy Browser yielded ~99% classified sequences among eight different phyla, particularly Cryptomycota, Chytridiomycota and Entomophthormycota (Figure 4). Surprisingly, compared to the bacteria, the fungal community remained quite different. As shown in Figure 4, the phylum Cryptomycota (98.25%) was particularly dominant in LL, while the phyla of Chytridiomycota and Entomophthormycota were the most abundant fungal phylum, accounting for 95.11% in the LL and 56.06% in the LG, respectively. A small proportion of sequences (0.38% for LL, 0.12% for LX and 0.39% for LG) were unclassified. The remaining fraction included Ascomycota, Basidiomycota and Blastocladiomycota. This is consistent with the study by Rojas-Jimenez et al. [35], which demonstrated that Chytridiomycota and Cryptomycota were most abundant phyla in the ice-covered lakes of the McMurdo Dry Valleys, Antarctica. The fungal community associated with the sediment of urban drinking water reservoirs was investigated by Zhang et al. [36], which was based on Roche 454 GS FLX pyrosequencing data. These results suggested that Chytridiomycota was the most abundant phylum in the sediments of source water reservoirs. Ascomycota and Basidiomycota were predominant in the Songhua Rive [37]. Aquatic fungi perform important roles in water nutrient recycling and regulating water quality, especially in fresh water lakes [8,35], rivers [13,19,37] and reservoirs [36]. More studies focused on fungal species should be undertaken in urban lake ecosystems in the future.

**Figure 5:**
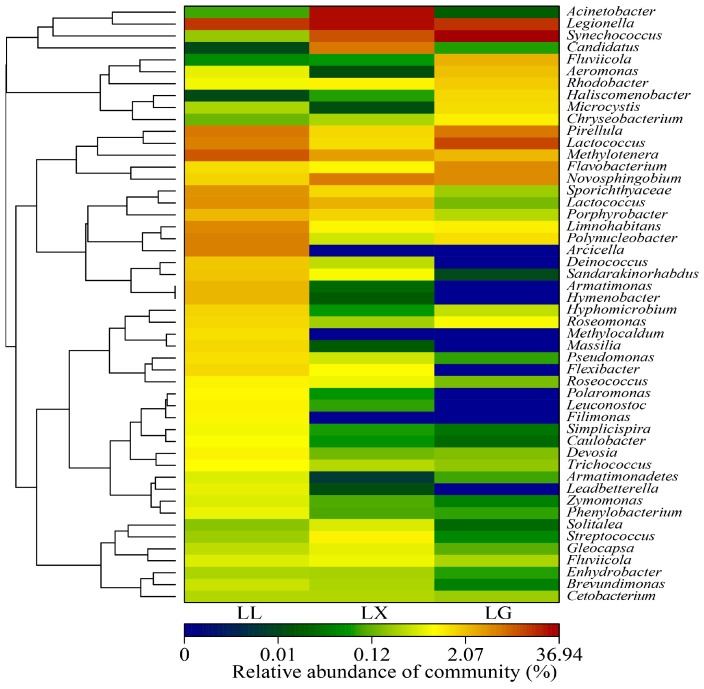
Heat map profile showing 50 representative predominant 16S rRNA gene-based sequence classified at the genus level using the Ribosomal Database Project (RDP) classifier database. LX, LG and LL represent Xingqing lake, Geming lake and Lianhu lake, respectively.

**Figure 6:**
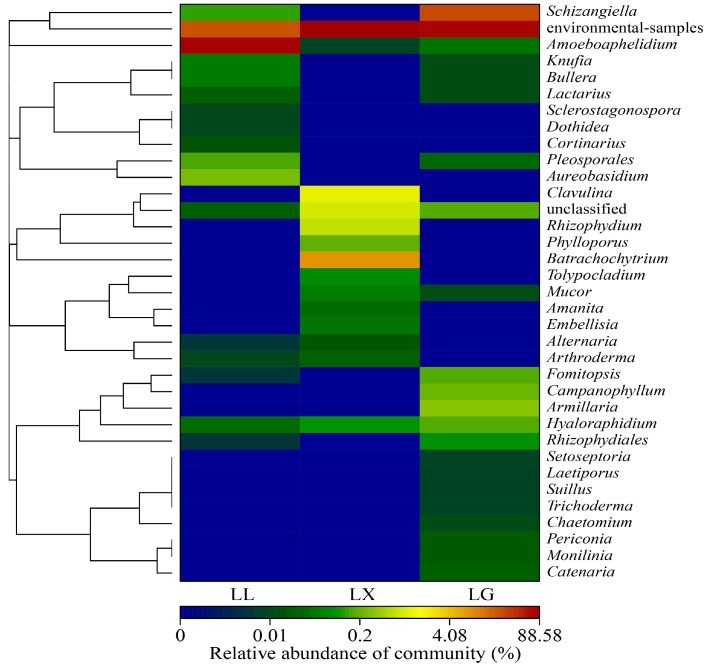
Heat map profile showing 35 representative predominant ITS gene sequence data based on classified at the genus level using the UNITE (http://unite.ut.ee) classifier database. LX, LG and LL represent Xingqing lake, Geming lake and Lianhu lake, respectively.

To better assess the different microbial communities in the three urban lakes, a more detailed profile of water bacterial community composition is illustrated by a hierarchically clustered heat-map at genus level. As shown in Figure 5, notable differences in the bacterial composition were observed between the three lakes. *Novosphingobium* sp., and *Synechococcus* sp. were recorded as the most dominant categories in the LX lake. LL contained the highest proportion of bacteria, with predominant genera of *Legionella* sp., *Methylotenera* sp., *Limnohabitans* sp. and *Arcicella* sp. (Figure 5). The water bacterial communities of recreational freshwater lakes were investigated by analysis with the Roche 454 Pyrosequencing technique. From this study, *Legionella* sp. and *Methylotenera* sp. were frequently observed in East Fork Lake, Delaware Lake and Madison Lake [38,39]. The bacterial communities in the recreational urban lakes play vital roles in both determining water quality and people’s health, such as *Pseudomonas* sp., *Legionella* sp. and *Shigella* sp., which have potential public health risks [39]. The water fungal communities at genus level of three urban lakes have changed significantly. As shown in Figure 6, the genera *Amoeboaphelidium* sp. (76.91%) was particularly dominant in LL, while *Batrachochytrium* sp. (8.20%), *Clavulina* sp. (1.10%) and *Rhizophydium* sp. (0.70%) were dominant in LX. Finally, 89.10% of sequences were unclassified. *Schizangiella* sp. (26.18%), *Armillaria* sp. (0.33%) and *Campanophyllum* sp. (0.23%) were the most abundant fungal genera in LG (Figure 6). The most abundant (approximately 72.50%) unclassified fungal group belonged to the environmental-samples-no rank. The microbial communities among three urban lakes were significantly distinct, because water quality parameters, such as water temperature, DO, organic matter and nitrogen compound concentrations, can shape the water bacterial and fungal community structure and diversity [40,41]. The previous study conducted by Luby et al. [42] reported that the domination phyla was Proteobacteria, Bacteroidetes and Actinobacteria in hot springs of Lake Magadi and Little Magadi in Kenya. Previous reports indicated that the genus *Rhizophydium* sp. (relative frequency 30.98%), *Apophysomyces* sp. (8.43%), *Allomyces* sp. (6.26%) and *Rhodotorula* sp. (6.01%) are common in the JINPEN reservoir, while *Elaphomyces* sp. (20.00%) and *Mattirolomyces* sp. (39.40%) were the dominant genus in Zhoucun and Shibianyu reservoirs. The fungal communities were more diverse than our findings [36]. The dominant fungal species in urban lakes were different to those in rivers. For example, *Mrakia* sp. and *Simplicillium* sp. were common species in Songhua River, China [37].

Urban lakes play an important role in supplying water systems for recreation and human activities, especially for children. In this study, we were surprised to find that several microbes were related to human diseases, such as infectious diseases, neurogenerative and even metabolic diseases (Table 5). *Flavobacterium* sp., *Legionella* sp. and *Streptococcus* sp. are harmful to people’s health [42,43,44]. Meanwhile, *Batrachochytrium* spp. was dominant in LX lake. *Batrachochytrium* spp. is a fungal pathogen. A previous report by Smith et al. [38] revealed that the fungal pathogen *Batrachochytrium dendrobatidis* can infect South African tadpoles and contributes to the decline of amphibians. Bandh et al. [45] assessed the presence of human pathogenic opportunistic fungi in the lake water in Dal Lake, Kashmir, India. The opportunistic fungal pathogens, including *Aspergillus*, *Candida*, *Penicillium*, *Cryptococcus*, *Fusarium*, *Rhizopus* and *Mucor*, were isolated in pure cultures. Approximately 8% isolates were positive for fungal infections (e.g., human skin infections). Pathogenic microorganisms re directly transmitted when contaminated urban lake water is consumed or contacted by human. The risk of diseases in the urban lakes increased with a higher relative abundance of pathogenic bacteria OTUs in the water (Table 5).

**Figure 7:**
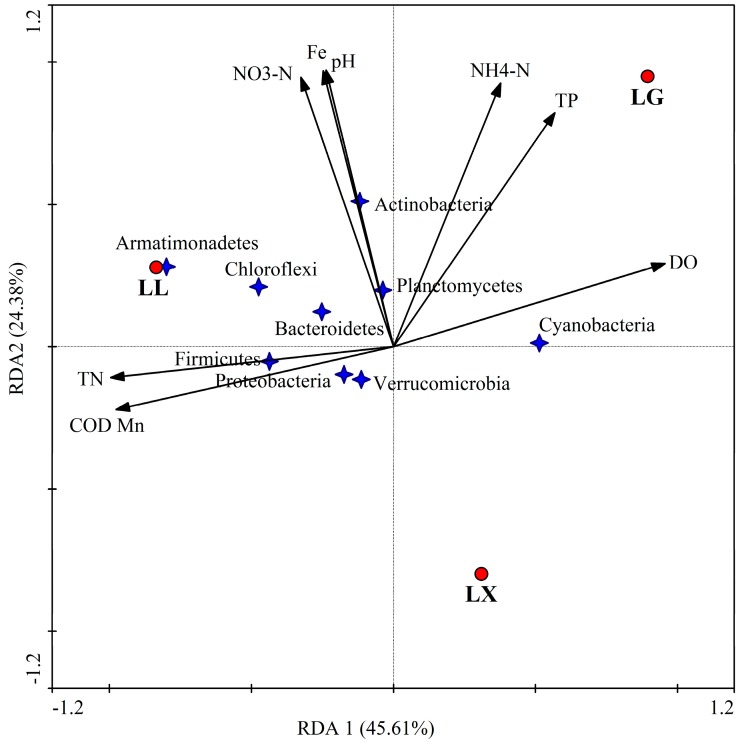
Redundancy analysis (RDA) of water bacterial communities in LX, LG and LL. RDA1 explained 45.61% and RDA2 explained 24.38% of the total variance. LX, LG and LL represent Xingqing lake, Geming lake and Lianhu lake, respectively. Water quality was found to be significantly correlated with bacterial community structure.

To assess the relationship between the water microbial community composition of the urban lakes and the water quality, redundancy analysis (RDA) was performed. The findings highlighted the dramatic differences among the urban lake samples. The results of the heat map analyses and RDA were in agreement. Both analyses revealed that the three urban lakes had distinct bacterial communities. As shown in Figure 7, ordination triplets of first two axes (RDA1 and RDA2) explained 69.99% of the total variance, respectively. RDA results indicated that the differences in bacterial communities were significantly correlated with the water quality parameters (Figure 7). The first axis of RDA1 was strongly positively correlated with water DO, TP and NH_4_^+^-N, but negatively correlated with COD_Mn_ and TN, which explained 45.61% of the total variance (*p <* 0.05, Monte Carlo based on 1000 permutations). DO had a positive effect on Cyanobacteria (*p <* 0.05). TN and COD_Mn_ had a positive effect on Firmicutes and Proteobacteria (*p <* 0.05, based on 1000 permutations). NH_4_^+^-N had a positive effect on Actinobacteria (*p <* 0.05, based on 1000 permutations). The second axis of RDA2 was strongly positively correlated with water pH, Fe and NO_3_^−^-N, which explained 24.38% of the total variance (*p <* 0.05, Monte Carlo based on 1000 permutations). NO_3_^−^-N had a positive effect on Armatimonadetes, Chloroflexi and Bacteroidetes (*p <* 0.05), which were apparently most dominant in LL.

**Figure 8:**
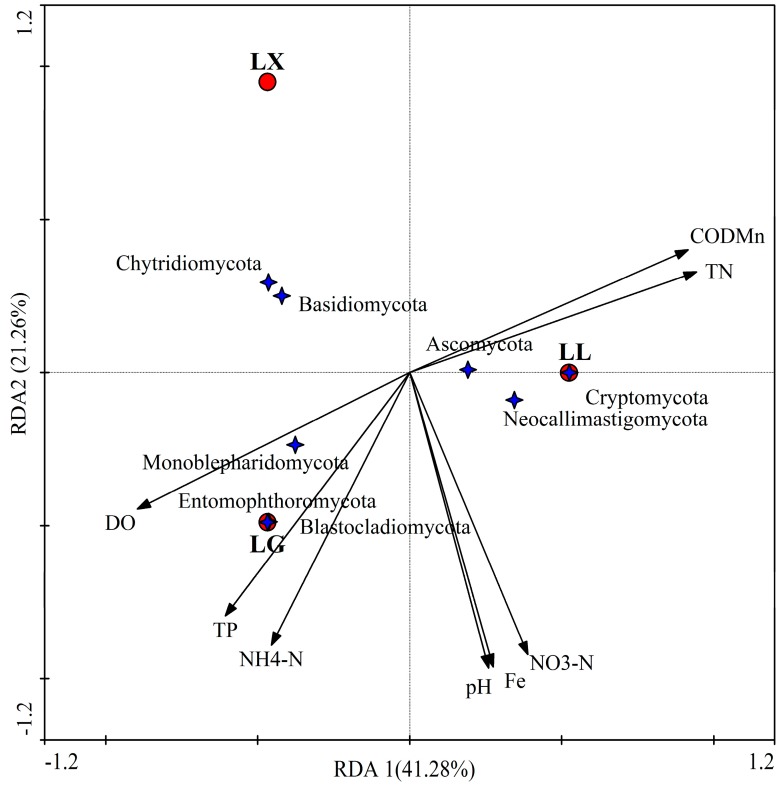
Redundancy analysis (RDA) of water fungal communities in LX, LG and LL. RDA1 explained 41.28%, and RDA2 explained 21.26% of the total variance. LX, LG and LL represent Xingqing lake, Geming lake and Lianhu lake, respectively. Water quality was found to be significantly correlated with fungal community structure.

As shown in Figure 8, for fungal communities, the first two RDA axes of water physical-chemical parameters explained 62.54% of the variation in fungal community structure. COD_Mn_ and TN were positively correlated with RDA1 (*p* = 0.02, Monte Carlo based on 1000 permutations). RDA2 was most strongly affected by TP, DO and ammonia nitrogen. Interestingly, previous studies suggested that the alkalization (pH of 8.5–9.0) of the lake water favors the growth of fungi that cause humans diseases [46]. Previous studies suggested that the water fungal community in the lakes or reservoirs were influenced by several factors, such as water physical properties (temperature and dissolved oxygen) [36], chemicals (chloride, sulfate and nutrients) [46] and microbial parameters (virus and algal blooms), which drive their increasing numbers in the urban lake. Therefore, the abundance and structure of water fungal communities particularly depend on the degree of pollution, the concentration of organic matter and algal biomass in lake [46]. Therefore, water fungi may be a reliable bio-indicator of lake water quality [36,46].

## 4. Conclusions

This study represents an attempt to assess the water quality and microbial community compositions in urban lakes. Using BIOLOG and Illumina Miseq sequencing techniques, the present study revealed diverse bacterial and fungal populations in three different urban lakes (Abbr. LL, LG, and LX). The results clearly showed that the water quality of these three lakes has significantly deteriorated. The highest total nitrogen (TN) was observed in LL with a value of 12.1 mg/L, which was two times higher than that of LG (*p <* 0.01). The COD_Mn_ concentration was 35.4 mg/L in LL. The highest *AWCD*_590nm_ was observed in LL. The water bacterial communities in the LL and LX had higher carbon source utilization ability. The lake water bacterial community was distributed among 14 different phyla, including Proteobacteria, Actinobacteria, Bactroidetes, Planctomycetes, Firmicutes, Armatimonadetes, Cyanobacteria, Chloroflexi, Verrucomicrobia, Fusobacteria, Chlorobi, Gemmatimonadetes, Acidobacteria and Spirochaeate. Cryptomycota was particularly dominant in LL, while the phylum Chytridiomycota and Entomophthormycota accounted for 95% in the LL and 56% in the LG, respectively. Heat map and redundancy analysis (RDA) highlighted the dramatic differences of water bacterial and fungal communities among the urban lakes. Meanwhile, the profiles of microbial communities were significantly correlated with the water quality parameters. We were also surprised to find that several microbes were related to human diseases, such as infectious diseases. Overall, these results represent an important advance in our understanding of the biological characteristics of urban lake water quality and enhancement of the assessment of the urban lake ecosystem inhabitants.

## Figures and Tables

**Table 1:** The geographical location of Xingqing lake (LX), Geming lake (LG) and Lianhu lake (LL) in Xi’an City, Shaanxi Province, China.

Urban Lakes	Latitude	Longitude	Surface Area (m^2^)	Built Year	Functions
LX	34°15′33″	108°58′39″	100,000	1965	Recreation
LG	34°16′42″	108°57′69″	20,000	1927	Recreation
LL	34°16′30″	108°55′59″	380,000	1916	Recreation

**Table 2:** Water quality parameters associated with Geming lake (LG), Lianhu lake (LL), and Xingqing lake (LX) in Xi’an City, Shaanxi Province, China.

Water Quality Parameters	LG	LL	LX	ANOVA
Temperature (°C)	24.1 ± 1.2A	25.3 ± 1.1A	23.6 ± 1.4A	NS
pH	9.6 ± 0.8A	9.4 ± 0.9A	8.3 ± 1.2A	NS
Dissolved oxygen (mg/L)	10.2 ± 0.02A	8.3 ± 0.01B	9.1 ± 0.04AB	*
NO_3_^−^-N (mg/L)	5.3 ± 0.4A	5.1 ± 0.5A	1.2 ± 0.07B	***
NH_4_^+^-N (mg/L)	2.8 ± 0.06A	1.9 ± 0.04B	1.5 ± 0.05B	*
Total nitrogen (mg/L)	5.6 ± 0.03C	12.1 ± 0.04A	8.4 ± 0.04B	**
Total phosphorus (mg/L)	0.21 ± 0.01A	0.08 ± 0.00B	0.06 ± 0.01B	**
COD_Mn_ (mg/L)	21.6 ± 2.3B	35.4 ± 2.9A	28.8 ± 1.6AB	**
Fe (mg/L)	0.08 ± 0.01A	0.07 ± 0.01B	0.01 ± 0.00C	**

Values shown as means and standard deviations (three replicates). Different capital letter represents statistical significance.* *p <* 0.05, ** *p <* 0.01 and *** *p <* 0.001 represent statistical significance using one-way ANOVA. NS represents no statistical significance.

**Table 3:** Functional diversity of water bacterial communities associated with Geming lake (LG), Lianhu lake (LL), and Xingqing lake (LX) in Xi’an City, Shaanxi Province, China.

Parameters	LG	LL	LX	ANOVA
*AWCD*_590nm_	0.49 ± 0.03C	0.90 ± 0.12A	0.67 ± 0.06B	**
Amino acids	0.17 ± 0.01C	0.74 ± 0.2A	0.57 ± 0.07B	***
Carboxylic acids	0.30 ± 0.08B	0.78 ± 0.2A	0.44 ± 0.03B	***
Carbohydrates	0.83 ± 0.1B	1.05 ± 0.2A	0.85 ± 0.05B	**
Amines	0.55 ± 0.03B	0.96 ± 0.1A	0.68 ± 0.02B	**
Phenolic compounds	0.41 ± 0.1B	0.67 ± 0.1A	0.68 ± 0.1A	*
Polymers	0.85 ± 0.1B	1.26 ± 0.2A	0.87 ± 0.1B	**
Richness diversity (*R*)	17 ± 1.20B	23 ± 0.9A	20 ± 2.3AB	*
Shannon’s diversity (*H’*)	3.50 ± 0.6C	5.4 ± 0.7A	4.48 ± 0.9B	*

Values shown as means and standard deviations (three replicates). Different capital letter represents statistical significance. * *p <* 0.05, ** *p <* 0.01 and *** *p <* 0.001 represent statistical significance using one way-ANOVA.

**Table 4:** Water bacterial and fungal community diversity index based on the Illumina Miseq sequencing data from Geming lake (LG), Lianhu lake (LL), and Xingqing lake (LX) in Xi’an City, Shaanxi Province, China.

Urban Lakes	Microbe	Reads	0.97 Level				
			OTUs	ACE	*Chao*1	Shannon Diversity (*H’*)	Simpson Diversity (*D*)
LG	Bacteria	13,598	231	254 (243, 276)	257 (242, 290)	3.52 (3.49, 3.55)	0.0766 (0.0742, 0.0790)
LL		26,252	380	393 (386, 407)	394 (385, 414)	4.55 (4.54, 4.57)	0.0198 (0.0194, 0.0202)
LX		22,892	283	302 (293, 321)	305 (292, 335)	3.48 (3.46, 3.51)	0.0854 (0.0833, 0.0875)
LG	Fungi	24,523	102	105 (103, 112)	103 (102, 108)	2.34 (2.32, 2.36)	0.1555 (0.1532, 0.1579)
LL		19,742	83	105 (93, 132)	98 (89, 126)	0.89 (0.87, 0.91)	0.6137 (0.6058, 0.6215)
LX		11,081	93	95 (93, 103)	94 (93, 103)	2.59 (2.56, 2.62)	0.1662 (0.1611, 0.1712)

**Table 5:** Relative abundance of OTUs related to human diseases.

Water Bacterial Species	LG	LL	LX	Potential Disease [42,43,44]
*Trichococcus* sp.	1	10	4	Intestinal infection
*Aeromonas* sp.	264	143	2	Diarrhea
*Brevundimonas* sp.	5	77	64	Intracranial infection
*Deinococcus* sp.	0	85	2	Infections
*Flavobacterium* sp.	597	338	207	Bloodstream Infections
*Kocuria* sp.	1	69	2	Respiratory tract infection
*Lactobacillus* sp.	0	66	2	Diarrhea
*Legionella* sp.	10	86	3	Pulmonary infection
*Pseudomonas* sp.	11	324	102	Respiratory system infection
*Streptococcus* sp.	0	32	12	Respiratory system infection

Numbers in the table represent OTUs in LL, LX and LG urban lakes.
